# Precursor B Cells Increase in the Lung during Airway Allergic Inflammation: A Role for B Cell-Activating Factor

**DOI:** 10.1371/journal.pone.0161161

**Published:** 2016-08-11

**Authors:** Konstantinos Samitas, Carina Malmhäll, Madeleine Rådinger, Patricia Ramos-Ramirez, You Lu, Tünde Deák, Maria Semitekolou, Mina Gaga, Margareta Sjöstrand, Jan Lötvall, Apostolos Bossios

**Affiliations:** 1 Krefting Research Centre, Department of Internal Medicine and Clinical Nutrition, Institute of Medicine, Sahlgrenska Academy, University of Gothenburg, Gothenburg, Sweden; 2 Cellular Immunology Laboratory, Division of Cell Biology, Center for Basic Research, Biomedical Research Foundation of the Academy of Athens, Athens, Greece; 3 7th Respiratory Medicine Dept. and Asthma Center, Athens Chest Hospital “Sotiria”, Athens, Greece; Telethon Institute for Child Health Research, AUSTRALIA

## Abstract

**Background:**

B cells, key cells in allergic inflammation, differentiate in the bone marrow and their precursors include pro-B, pre-B and immature B cells. Eosinophil progenitor cells increase in the lung after allergen exposure. However, the existence and possible role of B cell precursors in the lung during allergic inflammation remains elusive.

**Methods:**

A BALB/c mouse model of allergic airway inflammation was utilized to perform phenotypic and quantification analyses of pro-B and pre-B cells in the lung by flow cytometry. B cell maturation factors IL-7 and B cell-activating factor (BAFF) and their receptors (CD127 and BAFFR, BCMA, TACI, respectively) were also evaluated in the lung and serum. The effect of anti-BAFF treatment was investigated both *in vivo* (*i*.*p*. administration of BAFF-R-Ig fusion protein) and *in vitro* (colony forming cell assay). Finally, BAFF levels were examined in the bronchoalveolar lavage (BAL) of asthmatic patients and healthy controls.

**Results:**

Precursor pro and pre-B cells increase in the lung after allergen exposure, proliferate in the lung tissue *in vivo*, express markers of chemotaxis (CCR10 and CXCR4) and co-stimulation (CD40, CD86) and are resistant to apoptosis (Bax). Precursor B cells express receptors for BAFF at baseline, while after allergen challenge both their ligand BAFF and the BCMA receptor expression increases in B cell precursors. Blocking BAFFR in the lung *in vivo* decreases eosinophils and proliferating precursor B cells. Blocking BAFFR in bone marrow cultures *in vitro* reduces pre-B colony formation units. BAFF is increased in the BAL of severe asthmatics.

**Conclusion:**

Our data support the concept of a BAFF-mediated role for B cell precursors in allergic airway inflammation.

## Introduction

Asthma is a chronic airway disease that affects more than 300 million people worldwide [1]. Rather than a single disease entity, asthma is nowadays increasingly recognized as a syndrome embracing several clinical phenotypes that stem from different pathophysiological endotypes [2,3]. Depending on the inflammatory phenotype of asthma, distinct lymphocytic populations participate in different components of the immune response and can possibly be targeted therapeutically.

B cells are multifunctional lymphocytes that act as regulators of allergic inflammation. Apart from their role in humoral immune defense, B cells also act as potent antigen-presenting cells, produce numerous cytokines and regulate the way T cells mediate allergic inflammation [4–6]. B cells differentiate in the bone marrow (BM) from pluripotent haematopoietic stem cells (HSC) through the evolution of several precursor cell subsets that can easily be identified based on the expression of intracellular transcription factors and cell-surface molecules [6]. Early B lymphopoiesis and peripheral B cell maturation is regulated rigorously by several transcriptional factors and cytokines that act at specific time-points, such as the interleukin (IL)-7 and the B cell-activating factor (BAFF), respectively [6].

B cell progenitors are thought to be strictly located within the BM until they reach the stage of immature B cells and migrate to peripheral lymphoid organs for further maturation [6]. A similar approach was adopted for all other cell lines that differentiate in the BM. Our group and others have, however, recently demonstrated that eosinophil-committed progenitor cells can be recruited in the lung after allergen challenge, where they are able to further differentiate and proliferate [7–9], while anti-IL-5 treatment decreases their levels [10,11]. Moreover, circulating CD34^+^ progenitor cells were found to be increased in asthmatic patients [12]. To date, however, it is still unknown whether this also applies to B cells. Given the pronounced significance of B cells in initiating, establishing and maintaining allergic inflammation, it would be of great importance to investigate whether allergen challenge *per se* can induce the migration of B cell precursor subsets that are able of further maturation and proliferation *in situ* in the lung.

In this study we investigate the potential importance of such B cells precursor populations in the lung following allergen provocation using a well-established murine model of allergic inflammation. We identify and characterize specific B cell precursor subsets that increase in the lung after allergen exposure, express markers of functional activation and are able of chemotaxis and local proliferation. We further show *in vivo* and *in vitro* evidence of the participation of BAFF in the maturation and proliferation of these progenitor cells in the lung. Finally, pertinent to the human condition, we demonstrate that BAFF is also increased in the bronchoalveolar lavage fluid (BALF) of patients with severe asthma compared to patients with milder forms of the disease and healthy individuals.

## Materials and Methods

### Animals

BALB/c mice, 5 to 6 weeks old were purchased from Taconic (Ry, Denmark). All mice were kept under conventional or pathogen-free animal housing conditions and provided with food and water *ad libitum*. All surgery was performed under anesthesia, and all efforts were made to minimize suffering. The study was approved by the Ethical Committee for Animal Studies in Gothenburg, Sweden (permit no. 442–2008) and was carried out in strict accordance with corresponding guidelines.

### Allergen sensitization and allergen exposure

**Fig 1:**
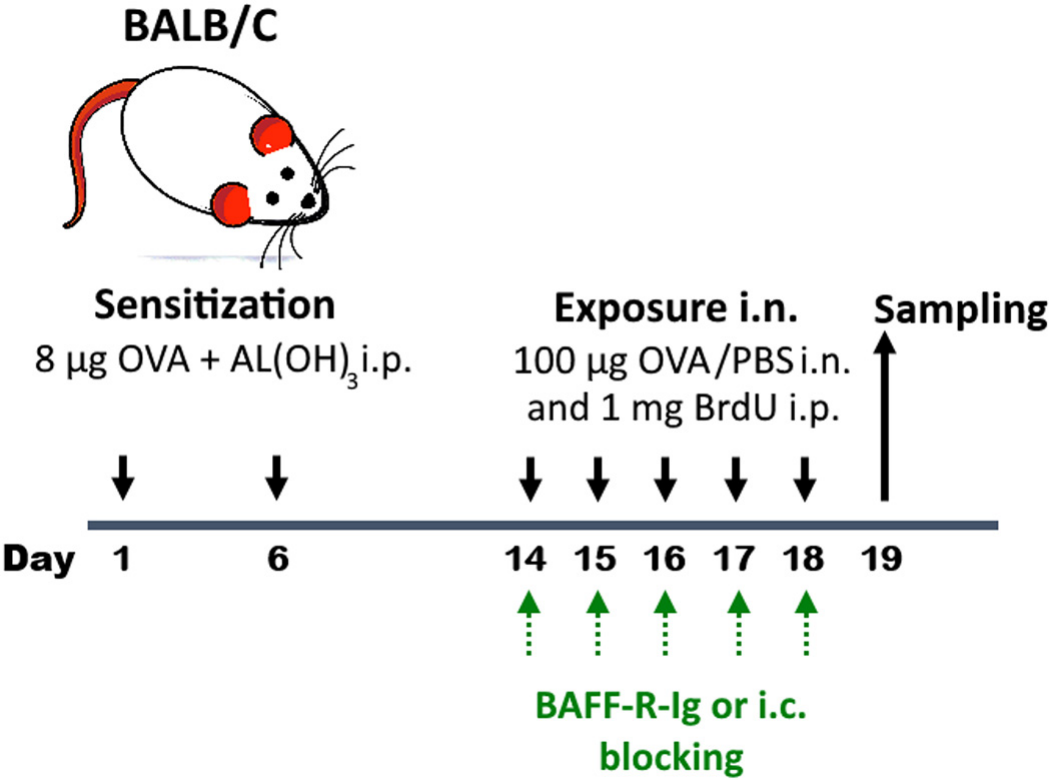
BALB/c mouse model of allergic airway inflammation. Schedule of OVA sensitization (8 μg each *i*.*p*.), OVA exposure (100 μg OVA in 25 μl PBS *i*.*n*. instillation each), BrdU administration (1 mg each *i*.*p*.) and tissue sampling in this model of allergic airway inflammation. All animals were sensitized to allergen, but control animals were exposed to PBS instead of OVA [13]. In another set of experiments designed to evaluate the role of BAFF, OVA sensitized mice received *i*.*n*. BAFF-R-Ig fusion chimeric protein (7 μg), which binds to its receptor (BAFFR) and blocks its action, or its isotype control protein, one hour prior to OVA exposure on five consecutive days, as indicated by green arrows.

The experimental allergen sensitization and exposure model utilized in this study has been previously described in detail [13] and is summarized in Fig 1. Briefly, mice were sensitized twice (Day 1 and Day 6) intraperitoneally (i.p.) with 8 μg chicken ovalbumin (OVA, Sigma-Aldrich, St Louis, MO, USA) bound to 4 mg aluminum hydroxide (Sigma-Aldrich) in phosphate buffered saline (PBS). On Day 14, mice were briefly anesthetized using isoflurane (Flurane^®^, Baxter, Deerfield, Ill, USA) and split in two groups; the exposure (OVA/OVA) received an intranasal (*i*.*n*.) administration of 100 μg OVA in 25 μl of PBS on five consecutive days, while the control (OVA/PBS) group received only PBS. In another set of experiments designed to evaluate the role of BAFF, OVA sensitized mice received *i*.*n*. instillations of BAFF-R-Ig fusion chimeric protein (7 μg; R&D Systems, Abingdon, UK), which binds to its receptor (BAFFR) and blocks its action [14], or its control protein, one hour prior to OVA exposure on five consecutive days, as indicated by green arrows (Fig 1).

### Evaluation of newly produced inflammatory cells

In order to label newly produced cells, all mice were administered 4 mg of the thymidine analogue BrdU (5-bromo-29-deoxyuridine, BrdU Flow Kits, BD Pharmingen^TM^, San Diego, Calif), as previously described [13]. BrdU was given at a dose of 1 mg in 0.25 ml of PBS by *i*.*p*. injection twice a day 7 h apart, on days 1 to 5 of allergen exposure.

### Sample collection/processing and preparation of lung single-cell suspensions

The collection of biological samples and their processing has been previously described [13] and is presented in detail in S1 Materials and Methods.

### Flow cytometry and gating strategy

Several antibodies or their isotype controls in various combinations were used (more information is available in S1 Materials and Methods and S1 Table). Cells subjected to surface staining were pre-treated with 4% mouse serum (DAKO) for 15 min to prevent unspecific binding and incubated with the planned antibody combination or their isotype controls for 30 min at 4°C. Surface staining with 7-AAD was also performed as a viability marker to exclude dead cells. Cells were then fixed in 2% paraformaldehyde and analyzed within 24 hours. Cells subjected to further intracellular staining were fixed using Cytofix/Cytoperm buffer (BD Biosciences) for 15 min and subsequently stained with the intracellular antibodies and/or BrdU/7-AAD (as proliferation markers) according to manufacturer´s protocol. Gating was first set on intact cells based on forward and side scatter characteristics. Analysis was performed following the Fluorescence Minus One (FMO) approach [15]. All flow cytometric analyses were performed using a BD FACSAria^TM^ and FACS Verse Flow Cytometers running FACS Diva version 6.0 and BD FACSuite Software respectively (BD Biosciences, San Jose, Calif) and analyzed with FlowJo Software^®^ (TreeStar Inc., Ashland, Ore).

### Mouse Pre-B Colony Forming Cell (CFC) Assay

Bone marrow (BM) cells from OVA sensitized and exposed mice were harvested and introduced in a mouse Pre-B Colony Forming Cell (CFC) assay using methylcellulose complete media for Pre-B Cells (HSC009; R&D Systems, Minneapolis, USA), according to the manufacturer’s instructions. The assay is based on the ability of hematopoietic progenitors to proliferate and differentiate into colonies in a semi-solid media in response to cytokine stimulation. Cells were cultured in the presence of complete media (containing IL-7) for 3 days. At day 4 recombinant BAFF (50 ng/ml, R&D Systems), BAFF-R-Ig fusion protein blocking BAFFR (100 ng/ml, R&D Systems) or its control protein was added to the cells. Colony Forming Units (CFUs) were counted at day 7.

### Human subjects

BALF samples used for the determination of BAFF levels were acquired from a previously conducted study, in which non-atopic healthy controls (HC) and patients with mild-moderate (MMA) and severe asthma (SA) were recruited and underwent bronchoscopy [16]. BALF samples from a total of 44, including 14 HC, 11 MMA and 19 SA were analysed. Subject characteristics are summarized in Table 1. More information about subject characteristics, definitions, patient treatment, bronchoscopy and sampling procedures can be found in S1 Materials and Methods.

**Table 1:** Subject characteristics.

	Control	MMA	SA
**Subjects (*n*)**	14	11	19
**Age (yrs)**	47 ± 2.5	49 ± 3.9	48 ± 2.2
**Males/females (*n*)**	7/7	2/9	3/16
**BMI**	26 ± 0.9	26 ± 1.5	30 ± 1.3
**FEV**_**1**_ **pred (%)**	88 ± 4.3	73 ± 5.8	68 ± 3.5^a^
**Atopy (*n*)**	0	6	16
**IgE (IU/mL)**	30 ± 31	95 ± 27	358 ± 91^b^^,^^c^
**LABA (*n*)**		9	19
**ICS (mcg/day)**^d^		620 ± 65	1770 ± 96
**OCS (n, mcg/day)**^e^		0	8 (8.8 ± 0.7)

Values are depicted as mean ± SEM. MMA: mild-to-moderate asthma; SA: severe asthma; FEV_1_: forced expiratory volume in 1 second, IgE: immunoglobulin E, LABA: long lasting beta agonists, LTRA: leukotriene receptor antagonists, ICS: inhaled corticosteroids, OCS: oral corticosteroids.

^**a**^*p* < 0.01 compared to control subjects

^**b**^*p* < 0.01 compared to control subjects

^**c**^*p* < 0.01 compared to MMA

^**d**^Beclometasone dipropionate CFC or equivalent dose, mean ± SEM

^**e**^Prednisolone equivalent dose, mean ± SEM

### IL-7 and BAFF measurements

The concentration of BAFF and IL-7 in the serum and supernatants of lung homogenates and BM of BALB/C mice, were quantified by ELISA (R&D Systems) with a limit of detection (LOD) of 4.3 pg/ml and 6.3 pg/ml, respectively, according to manufacturer’s instructions. Regarding the measurements in human samples, a specific BAFF ELISA kit was used (R&D Systems) with a LOD of 12 pg/ml. Detection limits were calculated by adding two standard deviations to the measured mean optical density of the zero standard replicates and calculating the corresponding concentration. All measured samples were above the limit of detection.

### Statistical analysis

Data are expressed as mean ± SEM (standard error of the mean), unless specified otherwise. Regarding mice data, statistical analysis was performed using Mann-Whitney U-test or unpaired T-test where appropriate, to determine the significant differences between groups. Group comparisons for isotype control and BAFF-R-Ig fusion protein treated mice were performed by means of two-way ANOVA with appropriate *post-hoc* analysis. In human data, normal distribution was assessed using the D’Agostino-Pearson normality test. Statistical analyses were carried out using a non-parametric analysis of variance. The Mann-Whitney U-test was used for two-group comparisons; whereas the Kruskal-Wallis one-way ANOVA accompanied by Dunn’s *post-hoc* correction was used for three-group comparisons. Quantitative analysis of unknown samples was performed by means of 5-parameter logistic nonlinear regression (5-PL). Statistical software was used for all data analyses (SPSS version 22, IBM) and graph preparation (Prism v6; GraphPad, San Diego, Calif, USA). A *p* value < 0.05 was considered significant.

## Results

### Allergic airway inflammation

**Fig 2:**
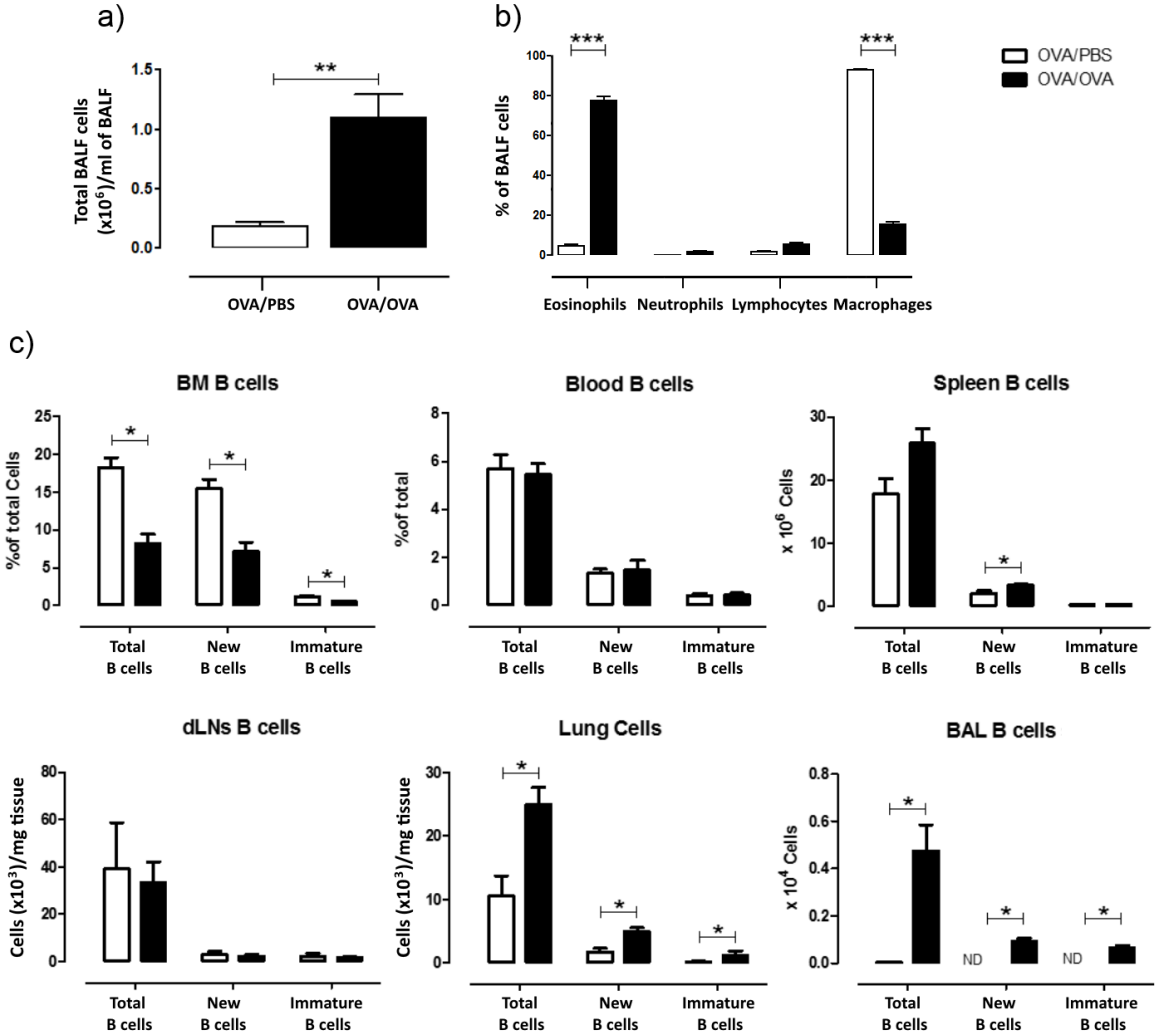
Establishment of allergic airway inflammation. (a) Total BAL fluid cell numbers were increased after OVA exposure. (b) BAL fluid eosinophils as a percentage of total BAL cells were increased after OVA/OVA exposure as compared to OVA/PBS exposure, while macrophages were decreased. (c) Total B cells (B220^+^SSC^low^), newly produced B cells during allergen exposure (B220^+^SSC^low^BrdU^+^) and immature B cells (B220^+^SSC^low^CD93^+^) in various biological compartments harvested upon allergen exposure. B cell precursor population significantly decreases in the BM after OVA exposure, and increases at the same time in the spleen, the lung and the BAL fluid. Data are presented as mean ± SEM, n = 5–8 mice/group, * *p* < 0.05, *i*.*p*.: intraperitoneally, *i*.*n*.: intranasally, *i*.*c*.: isotype control, ND: not detectable.

All animals were sensitized to OVA and exposed either to OVA (OVA/OVA) or to PBS (OVA/PBS) for five consecutive days, according to the protocol already presented in Fig 1. Allergen exposed animals showed an influx of inflammatory cells in the BALF (Fig 2A) and increased BALF eosinophilia (Fig 2B) compared to control PBS exposed animals, confirming the establishment of allergic airway inflammation. Similar results were obtained with respect to total lung cells (data not shown).

### B cell distribution during allergic inflammation

In order to assess the body distribution of B cells in the presence of established allergic airway inflammation, we examined total B cells (B220^+^SSC^low^), immature B cells (B220^+^SSC^low^CD93^+^) and newly produced B cells (B220^+^SSC^low^BrdU^+^) during allergen exposure in the following compartments: BM, serum, spleen, lung-draining mediastinal lymph nodes (dLNs), lung tissue and BALF (Fig 2C). We found that all B cell populations were decreased in the BM, while no differences were found in the serum or dLNs. Newly produced B cells were increased in the spleen. Interestingly, all three cell populations were increased in the BALF and lung tissue of OVA/OVA mice.

### B cell precursors in lung during allergic inflammation

**Fig 3:**
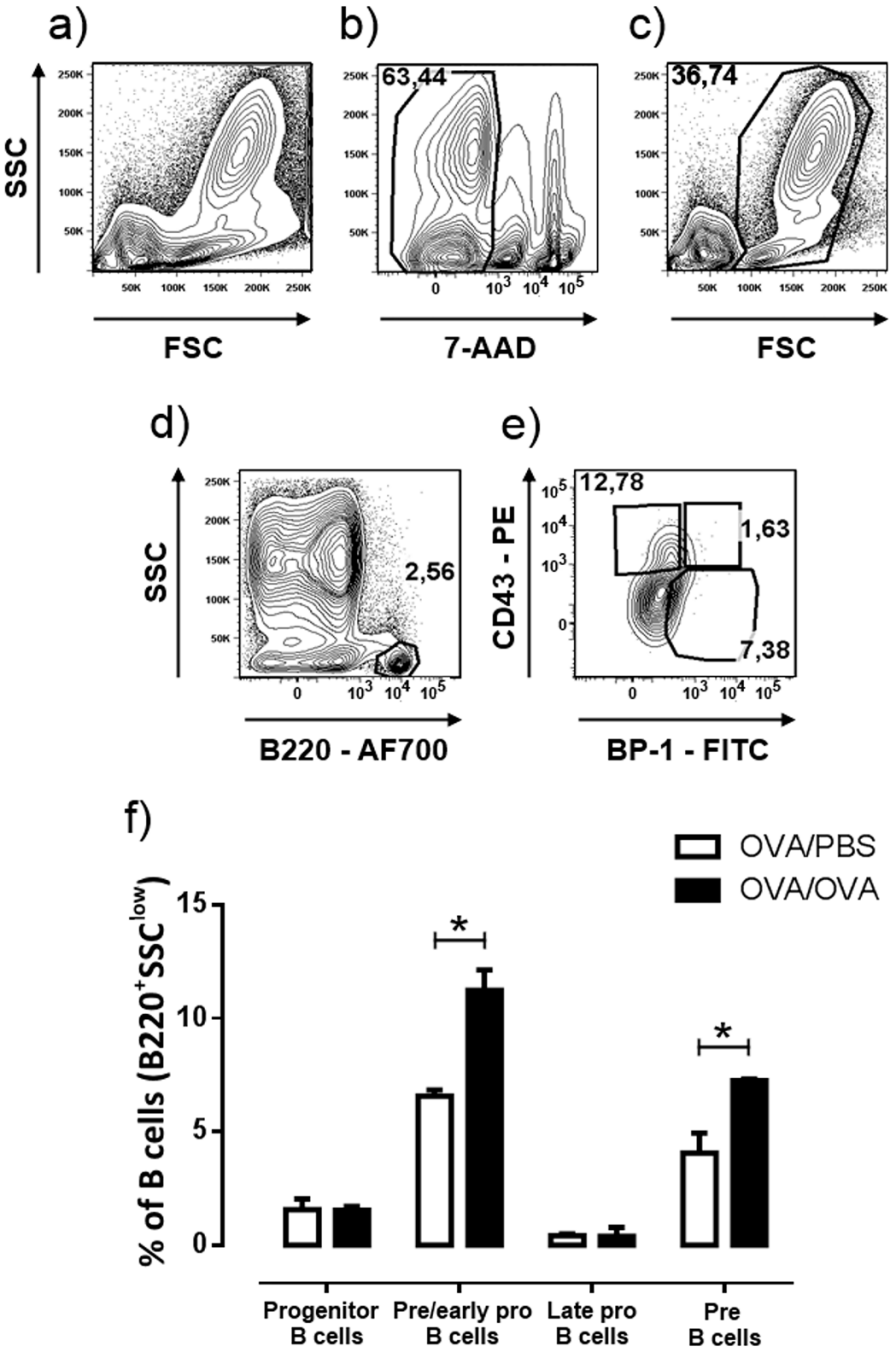
B cells precursors increase in the lung during allergic inflammation. Multipotent progenitors (B220^+^CD117^+^), pre/early pro-B cells (B220^+^CD43^+^BP-1^-^), late pro-B cells (B220^+^CD43+BP-1^+^) and (small) pre-B cells (B220^+^CD43^-^BP^-^1^+^), were evaluated. (a-e) A representative FACS plot analysis is presented (contour plots, 5% level with outliers). (a) Total lung cells as they are acquired in FACS. (b) Dead/not intact cells are excluded as they are 7-AAD positive. (c) Gating of intact leucocytes based in their morphological characteristics. (d) Gating of B mononuclear cells. (e) Gating of pre/early pro-B (CD43^+^BP-1^-^), late pro-B (CD43^+^BP-1^+^) and pre-B cells (CD43^-^PB-1^+^). (f) An increase of pre/early B and pre-B cells was observed upon allergen exposure. Data are presented as mean ± SEM, n = 5–8 mice/group, * *p* < 0.05.

As immature B cells increase in the BALF and lung tissue after OVA exposure, we explored the possibility that this population included B cell precursor subsets. We evaluated multipotent progenitors (B220^+^CD117^+^), pre/early pro-B cells (B220^+^CD43^+^BP-1^-^), late pro-B cells (B220^+^CD43^+^BP-1^+^) and (small) pre-B cells (B220^+^CD43^-^BP-1^+^), according to the expression of specific cell-surface molecules [6]. All the aforementioned B cell populations were found to be present in the lung tissue of both OVA and PBS exposed mice. However, pre/early pro-B cells and pre-B cells were found to be significantly increased during airway allergic inflammation, arguing in favor of their active influx to the lung (Fig 3). Our results on the B cell precursor populations examined were also verified by a new independent experiment using a new panel of antibodies (S1 Fig).

**Fig 4:**
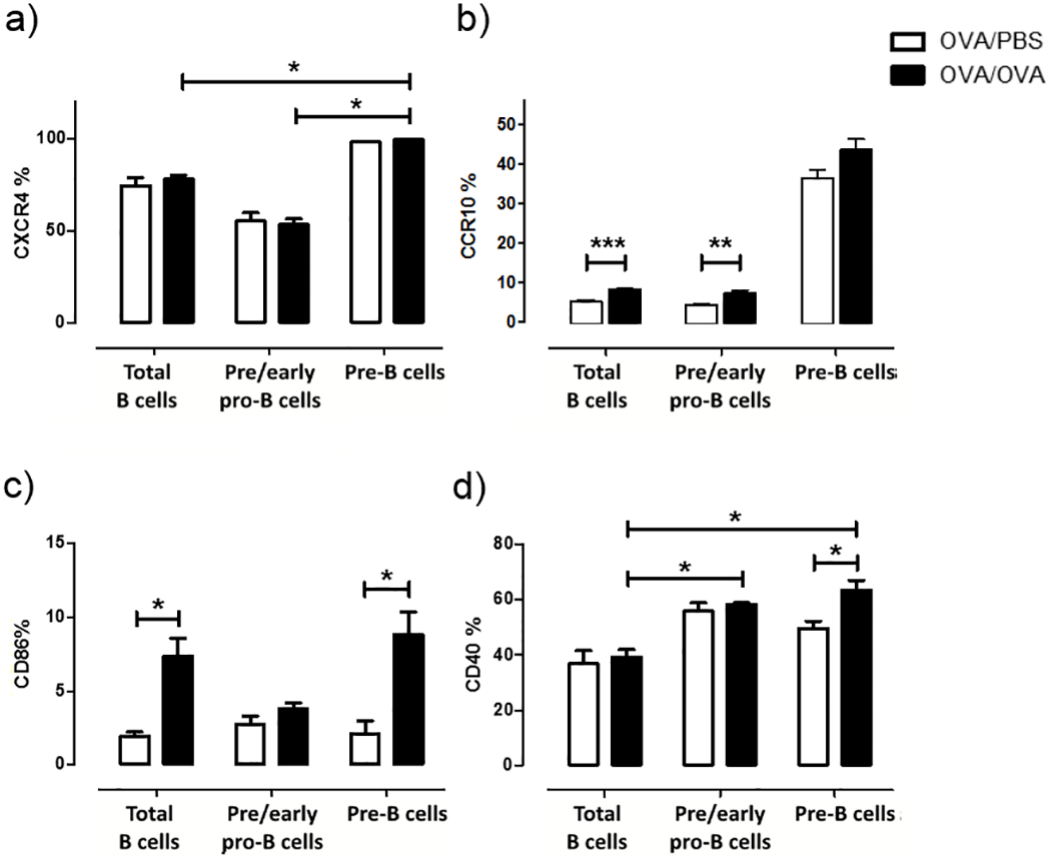
B cells precursors in the lung express markers of chemotaxis and functional activity. (a) CXCR4 expression is increased in pre-B cells (B220^+^CD43^-^BP^-^1^+^) compared to pre/early pro-B cells (B220^+^CD43^+^BP-1^-^) and total B cells (B220+SSC^low^). (b) CCR10 expression is increased in total B cells and pre-early pro-B cell precursors after allergen challenge. (c) CD86 and (d) CD40 activation markers are increased in pre-B cells in allergen challenged mice compared to controls and CD40 is increased in pre/early pro-B cells compared to total B cells. Data are presented as mean ± SEM, % refers to the percentage of positive cells out of total cells of each population, n = 5–8 mice/group, * *p* < 0.05.

Further characterization of the pro-B and pre-B cell populations was performed by expanding the range of key surface molecules’ expression not only in the aforementioned cell populations but also in total B cells, to be able to compare to baseline conditions. First, we evaluated their chemotactic ability by examining the expression of the chemokine receptors CXCR4 and CCR10, both crucial for B cell migration and chemotaxis to their homing tissues [17–19]. No differences were observed upon allergen challenge between PBS and OVA treated pre- and pro-B populations. However, we observed that CXCR4 expression increased significantly as B cells matured from pro-B cells to pre-B cells, when we compared cells in the same group i.e. OVA pro-B to OVA pre-B cells (Fig 4A). Interestingly though, a significant increase in the expression of CCR10 was observed not only in total B cells but more importantly in the pre/early pro-B cell precursor subset after allergen challenge (Fig 4B).

In order to examine the functional status of B cell precursor populations, we investigated the expression of CD86 and CD40 surface markers. Antigen presentation represents a main function for B cells, for which the CD86 and CD40 proteins are a prerequisite, as they provide co-stimulatory signals necessary for T cell activation and survival [20]. Pre-B, but not pre/early pro-B, cells presented increased expression of CD86 upon allergen exposure, on par with mature B cells (Fig 4C). Moreover, both pre/early pro-B cells and pre-B cells demonstrated increased levels of CD40 upon allergen exposure compared to total B cells (Fig 4D). The activation status of pre-B and pro-B cell populations was also evaluated with respect to CD69 expression level. Pre/early pro-B cells showed decreased expression of CD69 during allergic inflammation, while no differences were found in pre-B cells (data not shown).

### Lung B cell precursors express markers of apoptosis resistance and proliferate *in vivo* during allergic inflammation

**Fig 5:**
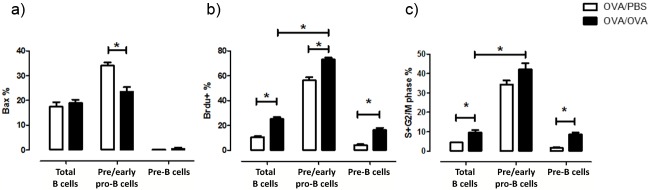
B cells precursors in the lung express markers of resistance to apoptosis and proliferation. (a) The apoptosis regulator Bax is downregulated in pre/early pro-B cells (B220^+^CD43^+^BP-1^-^) upon allergen exposure. (b) Newly produced total B (B220+SSClow), pre/early pro-B and pre-B cells (B220^+^CD43^-^BP^-^1^+^) increase in the lung upon allergen exposure, as designated by increased expression of the thymidine analogue BrdU (5-bromo-29-deoxyuridine). (c) Pre/early pro-B cells and pre-B cells proliferate *in-situ* during allergen exposure. Data are presented as mean ± SEM, % refers to the percentage of positive cells out of total cells of each population, n = 5–8 mice/group, * *p* < 0.05.

Subsequently, we investigated whether B cell precursor populations found in the lung tissue are resilient to apoptotic procedures by assessing the expression levels of the pro-apoptotic Bax and anti-apoptotic Bcl-2 transcription factors [21]. Pre/early pro-B cells showed decreased expression of Bax during allergic inflammation, while pre-B cells exhibited decreased Bax levels compared to total B cells and pre/early pro-B cells regardless of allergen exposure (Fig 5A). No differences were found in total B cells. Bcl-2 expression was less than 0.4% in all cell populations, without any significant differences upon allergen exposure (data not shown).

The quantification of newly produced B cell precursors upon allergen exposure was performed by measuring the incorporation of BrdU and 7-AAD into their DNA [13,22]. Both newly produced pre/early pro-B and pre-B cells increased during allergic inflammation. Importantly, the percentage of pre/early B cells that are newly produced was significantly higher compared to the percentage of total B cells that were newly produced (Fig 5B). We also evaluated the percentage of B cell precursors that were in a proliferative state (7AAD^++^/S+G2/M phase). Pre-B and total B cells showed increased proliferation in the lung during allergic inflammation (Fig 5C). Proliferating pre/early pro-B cells were significantly higher than total B cells.

### Lung IL-7 and B precursors IL-7R expression during allergic inflammation

**Fig 6:**
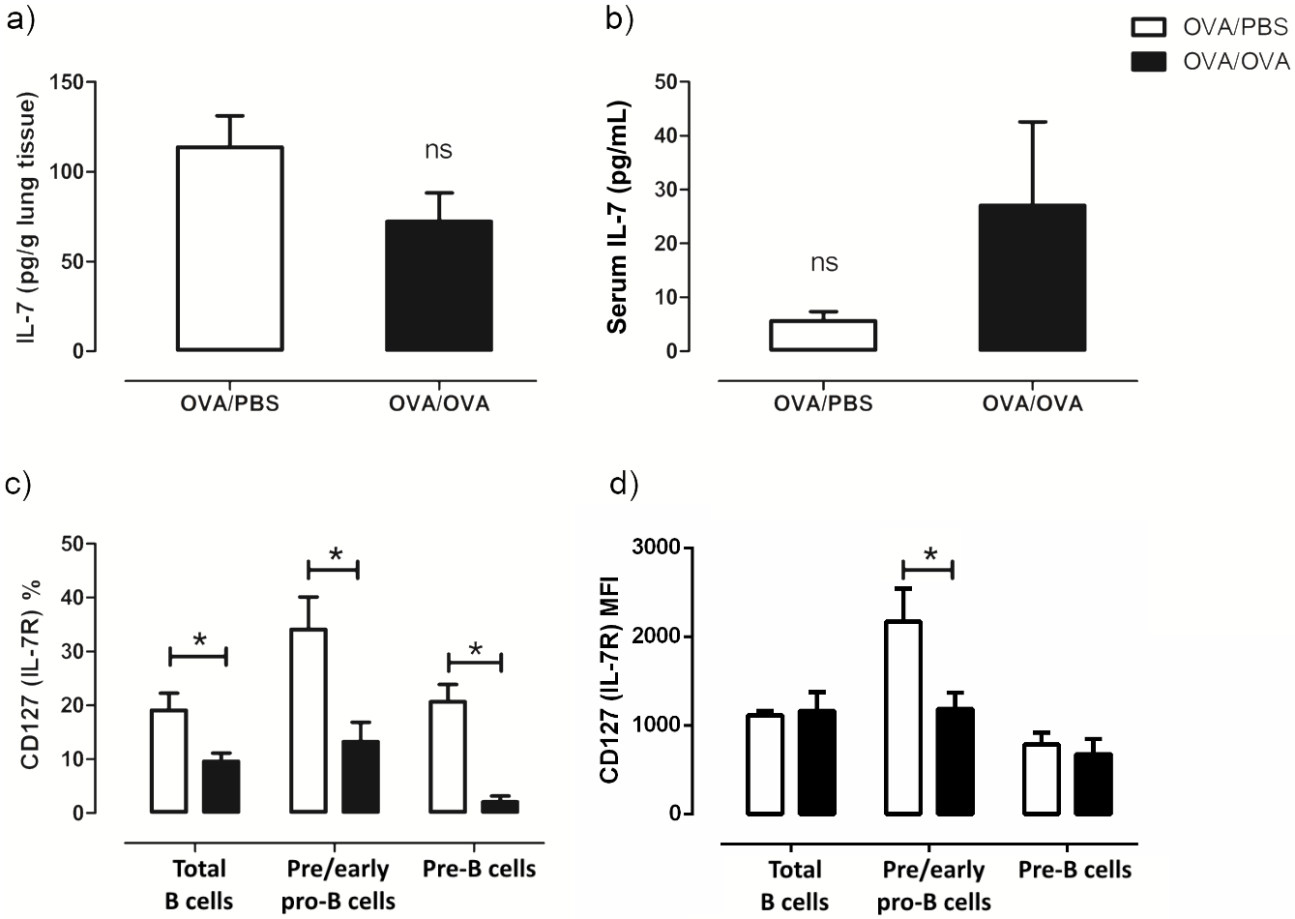
Lung and serum IL-7 and B precursors IL-7R expression during allergic inflammation. IL-7 levels in the a) lung and b) serum are not affected by allergen exposure. c) Expression of IL-7R was decreased upon allergic exposure. d) No differences were found in the intensity of the expression of the IL-7R in total B cells (B220+SSC^low^) and pre-B cells (B220^+^CD43^-^BP^-^1^+^), while in pre/early pro-B cells (B220^+^CD43^+^BP-1^-^) it was decreased during allergic inflammation. Data are presented as mean ± SEM, IL-7: interleukin-7, IL7R: interleukin-7 receptor, % refers to the percentage of positive cells out of total cells of each population, ns: not significant, n = 5–8 mice/group, * *p* < 0.05.

As IL-7 plays a very important role in B lymphopoiesis [6], we hypothesized that IL-7 is increased in the lung during allergen exposure and that B cells precursors will have increased expression of the corresponding IL-7 receptor (IL-7R). We measured IL-7 in the lung and serum to evaluate local and systemic changes. IL-7 was detected but was not affected by allergen exposure in either the lung (Fig 6A) or serum (Fig 6B). IL-7R (CD127) expression was decreased in total B, pre/early-pro B and pre-B cells upon allergic exposure (Fig 6C). The intensity of the expression of the IL-7R was significantly decreased in pre/early pro-B cells in OVA/OVA mice during allergic inflammation compared to control mice, while no differences were found in total B or pre-B cells. (Fig 6D).

### BAFF levels and BAFFR expression in B cell progenitors during allergic inflammation

**Fig 7:**
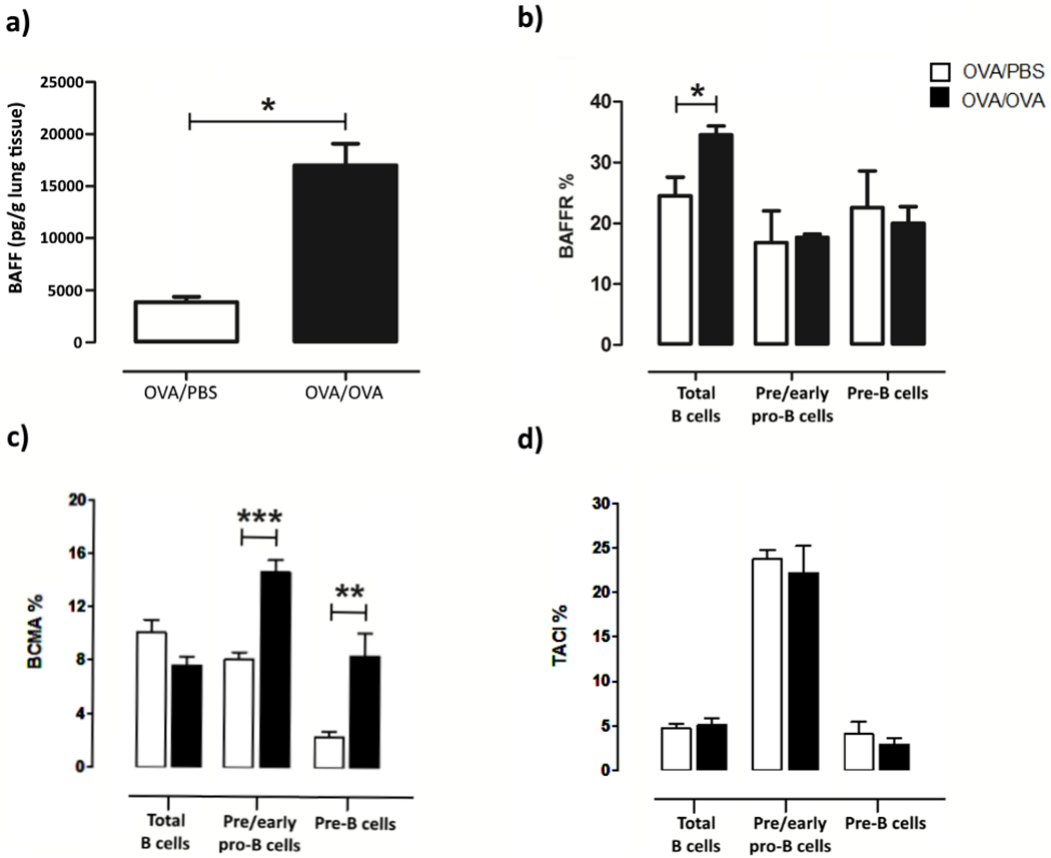
BAFF levels and BAFF receptors’ expression (BAFFR, BCMA, TACI) in B precursors BAFFR expression in the lung during allergic inflammation. (a) BAFF levels are increased during allergen exposure in the lung. (b) Total B cells (B220+SSC^low^), pre/early pro-B (B220^+^CD43^+^BP-1^-^) and pre-B cells (B220^+^CD43^-^BP^-^1^+^) express BAFFR and its expression increases in total B cells after allergen exposure. (c) Pre/early pro-B and pre-B cells express the BAFF receptor BCMA and its expression increases significantly in both B cell precursor populations. (d) Pre/early pro-B and pre-B cells also express the BAFF receptor TACI, however, no change in its expression is observed after allergen challenge. Data are presented as mean ± SEM, n = 5–8 mice/group, * *p* < 0.05, BAFF: B cell-activating factor, BAFFR: BAFF receptor, BCMA: B cell maturation antigen, TACI: transmembrane activator and calcium modulator and cyclophilin ligand interactor, % refers to the percentage of positive cells out of total cells of each population.

Since IL-7 or its receptor IL-7R was not found to be increased in lung tissue B cell progenitors during allergen exposure, we investigated the possible participation of other factors. BAFF is an important co-stimulator of B cell maturation, proliferation and function, although it has not been previously shown to be expressed in pre/early pro-B (B220^+^CD43^+^BP-1^-^) or pre-B cells (B220^+^CD43^-^BP^-^1^+^) [6]. We therefore examined BAFF levels in the lung, BALF and serum, as well as BAFFR expression in all cell populations. BAFF was not increased in the serum (S2 Fig), but was increased significantly in the BALF (S3 Fig) and the lung (Fig 7A) in allergen challenged mice. BAFFR expression was increased in total B cells but not in pre/early or pre-B cells (Fig 7B). Interestingly, pre/early pro-B and pre-B cells expressed the BAFF receptor BCMA and its expression increased significantly in both B cell precursor populations (Fig 7C). Moreover, pre/early pro-B and pre-B cells also expressed the BAFF receptor TACI, however, no change in its expression is observed after allergen challenge (Fig 7D).

### Effects of BAFFR blockage in the lung during allergic inflammation

**Fig 8:**
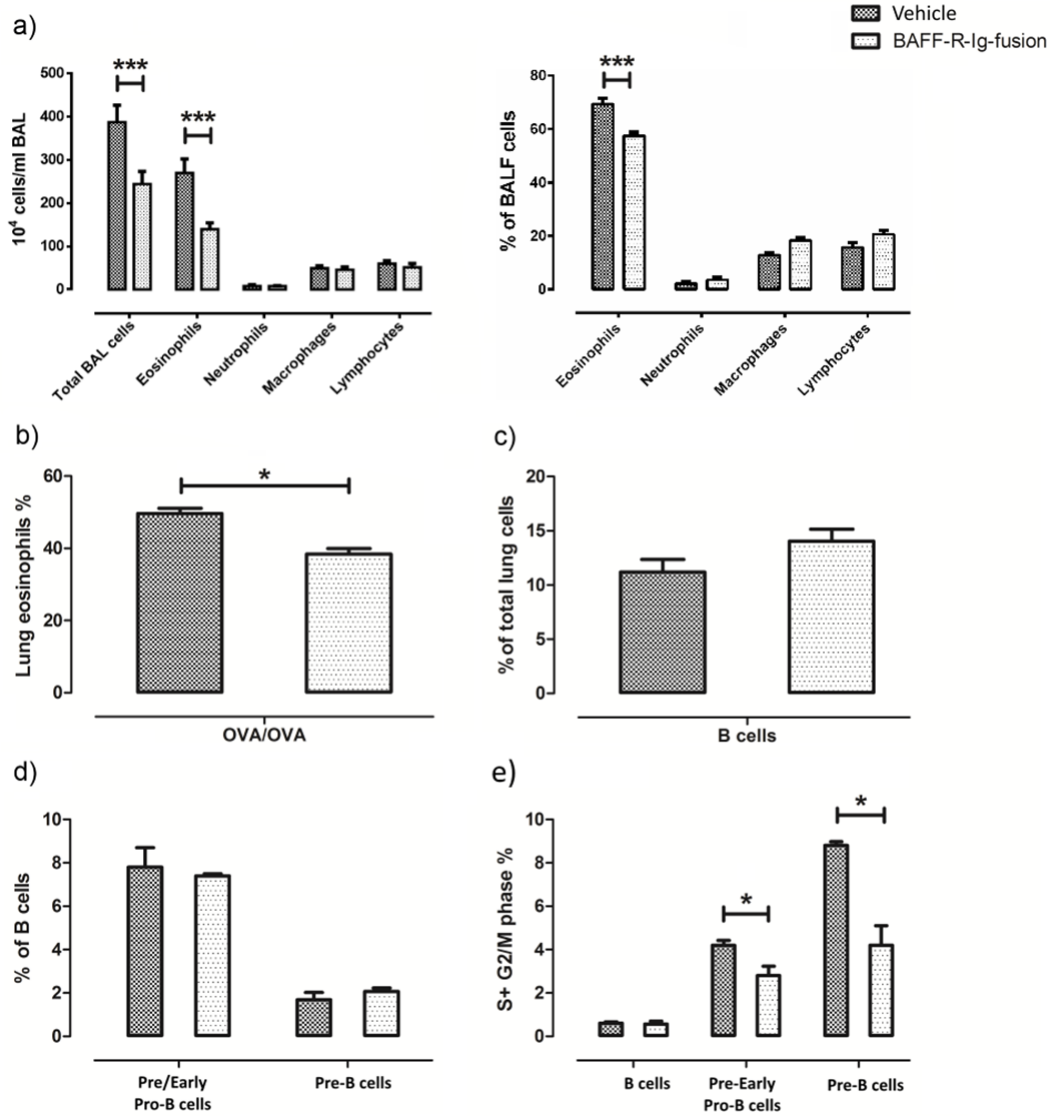
Effects of *in vivo* and *in vitro* blocking of BAFF in the lung. Administration of BAFF-R-Ig fusion protein *in vivo* in OVA sensitized and challenged mice resulted in decreased eosinophilic inflammation in the a) BAL fluid and b) lung tissue, compared to its control protein, c) while the total number of lung B cells or d) B cell precursor populations remained unchanged. e) However, blocking BAFF resulted in decreased numbers of pre/early pro-B and pre-B cells undergoing proliferation in the lung. Data are presented as mean ± SEM, * p < 0.05, *** p < 0.001, n = 6–8 per group.

The elevated BAFF levels in the lung of OVA challenge mice, the increased expression of its receptors BAFFR and BCMA in particular in total B cells and in pro and pre B cells after allergen challenge, argue in favor of a possible role for BAFF in the regulation of allergic inflammation and especially in B cells. To further test this hypothesis we used the same OVA-challenge mouse model and administered a BAFF-R-Ig fusion protein at specific time-points (indicated in green in Fig 1) that binds to BAFFR, blocking the binding of BAFF, and hinders further propagation of downstream signaling. This resulted in a significant drop of BALF (Fig 8A) and lung eosinophils (Fig 8B) despite allergen challenge compared to the control protein. No effect was found in total B cells (B220+SSC^low^) (Fig 8C) or in B cell precursors (Fig 8D). Interestingly, however, the percentage of lung pre/early pro-B (B220^+^CD43^+^BP-1^-^) and pre-B cells (B220^+^CD43^-^BP^-^1^+^) undergoing proliferation was also decreased after treatment with the BAFF-R-Ig protein (Fig 8E).

### Effects of BAFFR blockage in an *in-vitro* model of B cells lymphopoiesis

**Fig 9:**
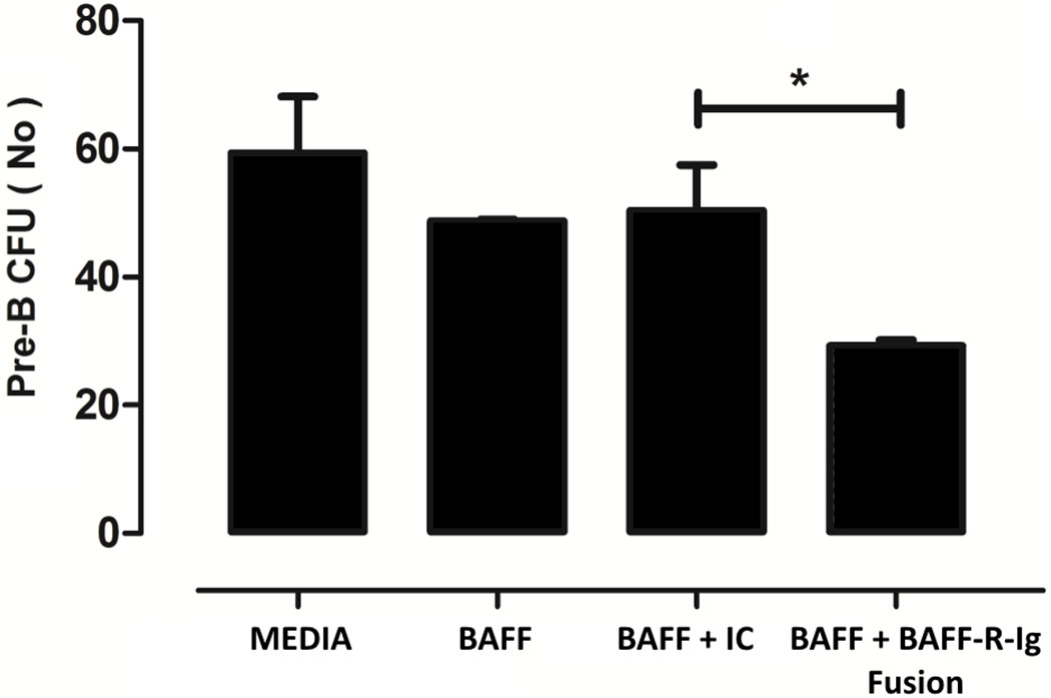
Effects of *in vitro* blocking of BAFF on B cell precursor development. Blocking BAFF in an *in-vitro* model of bone marrow cell culture causes developmental arrest, as shown by the decreased number of pre-B cells colony forming units. Data are presented as mean ± SEM, * *p* < 0.05, results are representative of two independent experiments. BAFF: B cell-activating factor, I.C.: isotype control.

In order to further explore the specific role of BAFF with respect to the proliferation and maturation of the B cell precursor subsets we had previously discovered in the lung after allergen challenge, we used BM cells from OVA sensitized and exposed mice and placed them in a mouse Pre-B Colony Forming Cell (CFC) assay. The assay is based on the ability of hematopoietic progenitors to proliferate and differentiate into colonies in a semi-solid media in response to cytokine stimulation. Several conditions were engaged including the presence of BAFF and the BAFF-R-Ig fusion protein. We found a significant reduction of pre-B Colony Forming Units (CFU) in BM cells treated with BAFF-R-Ig fusion protein (Fig 9).

### BAFF expression in the BALF of patients with asthma

**Fig 10:**
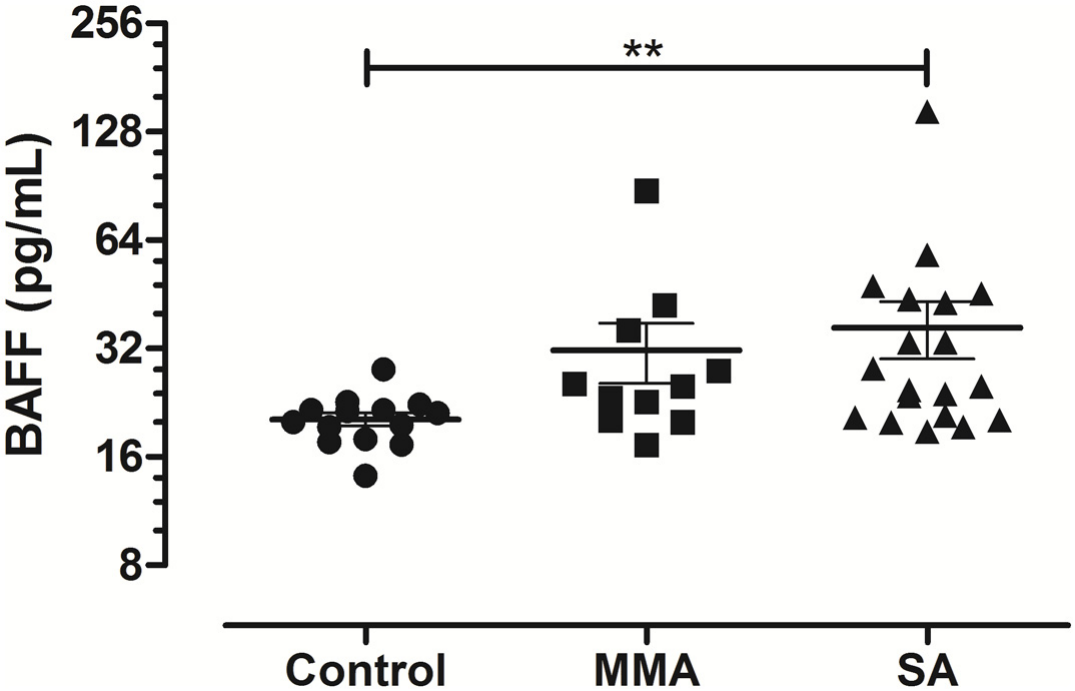
BAFF levels are increased in the BALF of patients with severe asthma. BAFF levels were evaluated in the BALF of patients with asthma of varying severity and healthy control volunteers. Data are presented as mean ± SEM, * *p* < 0.05, BAFF: B cell-activating factor, BALF: bronchoalveolar lavage fluid, SA: severe asthmatics, MMA: mild-moderate asthmatics.

After establishing that BAFF is upregulated and involved in the regulation of progenitor B cells in the lung after allergen challenge, the next step would be to investigate whether this would be relevant to the human condition. We explored the possibility that BAFF is also upregulated in human asthma and examined BAFF levels in the BALF of healthy controls as well as patients with mild-moderate and severe asthma. BAFF was incrementally increased with disease severity reaching statistically higher levels in severe asthmatics compared to healthy controls (Fig 10). BAFF levels in the BALF also correlated with the body mass index (BMI) of asthmatic patients (*p* = 0.018, r = 0.44; S4 Fig). No association was observed between BAFF levels and oral corticosteroid treatment (S5 Fig).

## Discussion

The findings of this study uncover a newly recognized role for progenitor B cells during allergic airway inflammation. We identify, for the first time, increased numbers of specific progenitor B cell subsets (pre/early pro- and pre-B cells) in the lung after allergen challenge. Moreover, we show that these B cell precursors are capable of proliferation and express markers of resistance to apoptosis, functional activity and chemotaxis, whose expression is increased upon allergen challenge. We also demonstrate that B cell precursors also express, apart from BAFFR, the other two BAFF receptors BCMA and TACI, and that BAFF upregulation plays a role not only in establishing eosinophilic allergic inflammation after allergen challenge, but also in the proliferation and, possibly, further differentiation of these newly found B cell progenitors in the lung. Finally, pertinent to the human condition, we demonstrate higher BAFF concentrations in the BAL of patients with severe asthma compared to patients with milder forms of the disease and healthy individuals.

Our view on progenitor cells has changed over the last few years. We have recently shown the presence of CD34^+^IL5^+^ cells in the bronchial tissue of asthmatics [23] and CCR3^+^CD34^+^ eosinophil lineage-committed cells in the lung after experimental allergen challenge [9], possibly mobilized from the periphery [12]. The accumulation of B cells within the airways shown in this study is most likely a combination of migration, prolonged survival and increased production of these cells in the bone marrow. Although *de novo* differentiation from pre-existing airway mesenchymal cells cannot be excluded, our data suggest rapid cell recruitment from the bone marrow reservoir to the periphery and target organs, as B cells decrease in the bone marrow after OVA challenge and increase in all other tissue compartments. In accordance with this hypothesis, we demonstrate that lung B cell precursors have the potential of responding to chemotactic stimuli, as they not only express the chemokine receptors CXCR4 and CCR10 on their surface, but also significantly increase CCR10 expression upon allergen exposure. CXCR4 controls immature B lymphocyte egress from the bone marrow [24] and is implicated in the lung-homing of endothelial progenitor cells in experimental asthma [25]. CCR10 has been found to play key role in plasma-cell homing [26]. Importantly, the CCR10 ligand, CCL28, is also increased upon allergen exposure [27], and can also serve as a ligand for CCR3, a major chemokine receptor for eosinophil progenitor cells [28]. As other chemokines, such as CXCL13, CCL19 and CCL2, are also central for B cell attraction in lungs [29], expression of their respective receptors at the surface of pro- and pre-B cells could give further details concerning their chemotactic profile.

B cell precursors in the lung also expressed the cellular activation markers CD86 and CD40, suggesting that they are functionally active. Both CD40 and CD86 play a main role in T cell activation and differentiation through CD28 and CD40L, respectively [30]. Interestingly, both pre/early pro B cells and pre B cells express more CD40 molecules per cell compared to B cells, arguing in favor of a more active role in B-T cell interaction. In accordance with our findings, innate pro-B cells were recently demonstrated to produce cytokines and regulate autoimmune effector T cells in response to IFN-γ [31], while pre-B cells are also capable of expressing CD2, CD25 and MHC class II surface molecules [32]. It is, therefore, possible that precursor B cells contribute to the T_H2_ bias in allergic inflammation.

CD86, specifically, is expressed in DCs as well in B cells and is upregulated upon antigen stimulation, preferentially inducing T_H2_ mediated inflammation. Asai-Tajiri and colleagues managed to experimentally attenuate allergic inflammation, including eosinophilia, IgE and T_H2_ cytokine expression in lung upon OVA-exposure, by blocking CD86 locally with siRNA [33]. The increased expression of CD86 we observed in pre B cells may also be related to their progression to more mature B cell subsets, as CD86 signaling has been found to participate in B cells differentiation [34].

Unexpectedly, expression of CD69 was decreased in pre/early pro-B cells upon allergen exposure. As CD69 expression is associated with increased apoptosis in eosinophils [35], the observed decline in CD69 expression may denote resistance to apoptosis. This hypothesis is also supported by our observation that pro-B cells demonstrate lower Bax expression, a well-known pro-apoptotic protein, indicating increased cell survival [36]. Moreover, lung eosinophilia during allergic inflammation may contribute to pro-B cell survival, as eosinophils have been recently demonstrated to promote B cell survival [37]. Studies with other apoptotic markers, such as cleaved caspase-3, may provide further information regarding the apoptotic status of pro-B cells found in the lung.

The bronchial tissue is admittedly a less friendly environment than the bone marrow, when it comes to progenitor cells. For B cell precursors to survive and further differentiate *in situ*, it is mandatory that certain factors are present in the lung, apart from increased eosinophilia. IL-7 constituted a plausible candidate, considering its vital role during early B cell differentiation and further pre-B cell survival and maturation [6,38]. The fact that the expression of both IL-7 and its receptor is unaltered (or even decreased in the case of IL-7R) was rather unexpected, but may be attributed to the possibility that these specific B cell precursor subsets have reached a maturation point where IL-7 is not needed. This notion is also supported by the down regulation of IL-7R we observed in all B cells populations and also the intensity of IL-7R expression we found in pre/early pro-B cells. The involvement of other survival/maturation factors is also possible.

BAFF was the next likely candidate to investigate, as it is generally considered a key factor for B cell survival, maturation and subset development that acts downstream of IL-7 [6,39]. We show that BAFF is highly upregulated in the lung after allergen exposure and that its receptors, BAFFR, BCMA and TACI are also expressed on B cell precursors, which constitutes a novel finding of this study. Importantly, we demonstrate for the first time that BCMA expression in pre/early pro B cells is significantly increased after allergen exposure, which, in combination with BAFF’s synchronous upregulation, argues in favor of an important role of the latter in the further development of these cells. In accordance with our findings, it has been recently shown that about 40% of immature B cells in the bone marrow also express BAFFR and that its expression correlates with B cell development and positive selection [40]. Furthermore, blocking BAFF signaling decreased B cell precursor proliferation, ameliorated airway allergic inflammation *in vivo*, and inhibited further pre-B cell differentiation *in vitro*. Collectively, our data support BAFF’s involvement in the regulation of B cell precursor biology not only in the bone marrow [40], but also in the lung.

The notion that BAFF has a regulatory role in allergic inflammation is further supported by our novel finding that it has a direct effect on eosinophils, which is in accordance with previous reports implicating BAFF with asthma, airway hyperresponsiveness and impaired lung function [41,42]. It has been suggested that BAFF is induced in the airways by allergen exposure and contributes to local class-switch recombination and immunoglobulin synthesis by B cells [43]. We provide evidence of an additional role for BAFF in the regulation of further maturation of B cell precursors in the challenged airways. Moreover, in accordance with previous reports of increased BAFF levels in the BALF of allergic non-asthmatic subjects after segmental allergen challenge [44], we show increased BAFF levels in the BALF of severe asthmatics, suggesting that BAFF is possibly involved in the pathogenesis of a persistent asthmatic airway inflammation. Although asthma treatment and especially oral corticosteroids may influence the expression levels of several cytokines, we did not observe any difference regarding the concentration of BAFF in the BALF of asthmatics threated with oral corticosteroids compared to those that were not. Finally, the observed correlation with patients’ BMI further adds to previous reports of BAFF acting as an adipokine in obesity related severe asthma, along with leptin and adiponectin [45,46].

An unexpected finding of our study is that immature B cells are also present in the lung tissues of control mice, although to a much lesser extent compared to OVA challenged mice. It is possible this could be attributed to sensitization alone [47] or to the possible circulation/accumulation of immature B cells in the airways even at steady state, as it is also observed in the intestinal mucosa where precursor B cells recombine their B cell receptor (BCR) [48]. Given its continuous exposure to antigenic stimuli, it is possible that the lung also acts as an organ favoring BCR diversification *in situ* and precursor B cells in the lung can be a source of allergen specific IgE [49].

This study presents certain limitations. The use of alum sensitization has been related to the activation of certain cell types, mostly eosinophils, monocytes and neutrophils, with less pronounced effects on B cells [50], however such an interaction cannot be excluded. Moreover, the aim of this study was to investigate the possible existence of B cell precursors in the lung upon allergen exposure, characterize their phenotypic profile and consider probable chemotactic/survival/maturation factors involved in their regulation. An in-depth characterization of their ability for activation, proliferation and survival was not in the scope of this study. The possible involvement of other factors beyond IL-7 and BAFF, such as IL4 and APRIL, was not investigated.

Taken together our data argue in favor of a novel role for BAFF in the regulation of allergen-driven accumulation, proliferation and, possibly, further differentiation of B cell precursor cells in the lung. Given the importance of B cells in antigen presentation and the preservation of allergic disease, it is possible that these B cell progenitors are involved in the initiation of the allergic response and that the development of therapeutic strategies targeting their differentiation in situ could potentially block the allergic response at its very onset. Further research is needed to fully elucidate the role of this newly discovered B cell precursor population in the challenged lung.

## Supporting Information

S1 Fig A new Flow Cytometry experiment was performed in order to confirm the existence of the main precursors B cell populations showed in Fig 2. Lung cells from an OVA/OVA mouse were processed and stained with specific antibodies and acquired in FACS Verse. A representative FACS plot analysis of an OVA/OVA mouse is presented (contour plots, 5% level with outliers). (a) Total lung cells as they are acquired in FACS. (b) Dead/not intact cells are excluded as they are 7-AAD positive. (c) Gating of peripheral blood mononuclear cells (PBMC) based in their morphological characteristics. (d) Excluding the doublets (gating in singles, FSC-A *vs* FSC-H +ve) cells, (e) gating in B220^+^ B cells, (f) & (g) Using the FMO (Fluorescence-minus-one) approach quadrant gates were set on CD43 & BP-1 background expression following by (h) gating of pre/early pro-B (CD43^+^BP-1^-^) and small pre-B cells (CD43^-^BP-1^+^).
(TIF)

S2 Fig Concentration of BAFF in the serum of OVA challenged mice compared to control mice. (TIF)

S3 Fig BAFF levels are increased in the BALF of OVA challenged mice compared to control mice. (TIF)

S4 Fig BAFF levels in the BALF correlated with the body mass index (BMI) of asthmatic patients. (TIF)

S5 Fig Concentration of BAFF in the BALF of asthmatics threated with oral corticosteroids compared to those that were not. (TIF)

S1 Materials and Methods Supplementary information regarding the Materials and Methods section. (DOCX)

S1 Table Antibodies used for Flow Cytometry. (DOCX)

S2 Table Stepwise differentiation of HSCs to immature B cells in the bone marrow, depicting the expression of cell-surface molecules according to their developmental stage and underlining the surface markers used in the flow cytometry to identify B cell subtypes. B cell precursor subsets as well as the markers used for their identification are highlighted in yellow. ** p < 0.01.
(DOCX)
