# Supplementary figures and images for: Precursor B Cells Increase in the Lung during Airway Allergic Inflammation: A Role for B Cell-Activating Factor
Source: PLoS One. 2016 Aug 11;11(8):e0161161. doi: 10.1371/journal.pone.0161161 (PMC4981371; doi:10.1371/journal.pone.0161161)

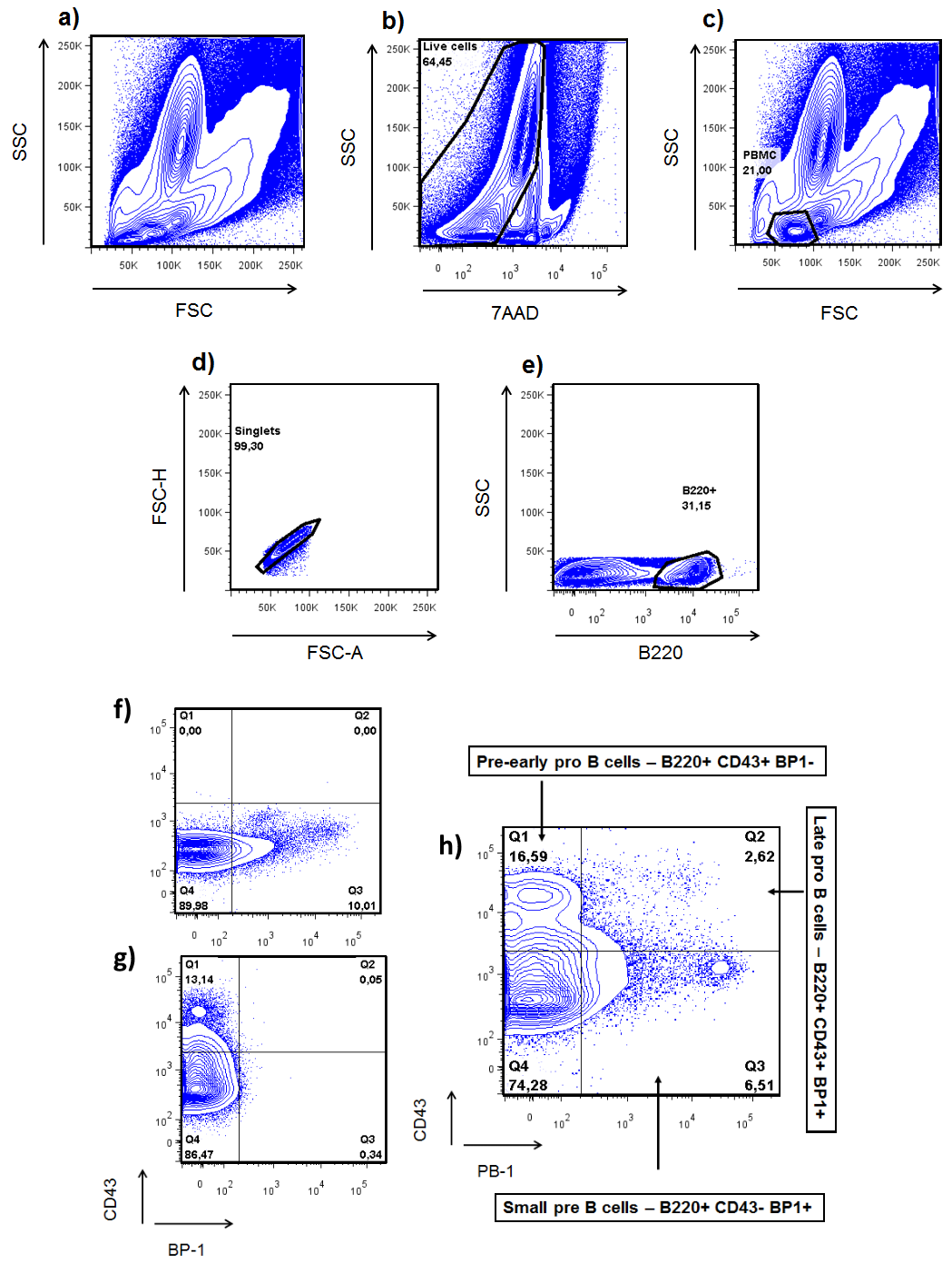

Supplement: S1 Fig — Lung cells from an OVA/OVA mouse were processed and stained with specific antibodies and acquired in FACS Verse. A representative FACS plot analysis of an OVA/OVA mouse is presented (contour plots, 5% level with outliers). (a) Total lung cells as they are acquired in FACS. (b) Dead/not intact cells are excluded as they are 7-AAD positive. (c) Gating of peripheral blood mononuclear cells (PBMC) based in their morphological characteristics. (d) Excluding the doublets (gating in singles, FSC-A vs FSC-H +ve) cells, (e) gating in B220+ B cells, (f) & (g) Using the FMO (Fluorescence-minus-one) approach quadrant gates were set on CD43 & BP-1 background expression following by (h) gating of pre/early pro-B (CD43+BP-1-) and small pre-B cells (CD43-BP-1+). (TIF) [file pone.0161161.s001.tif]

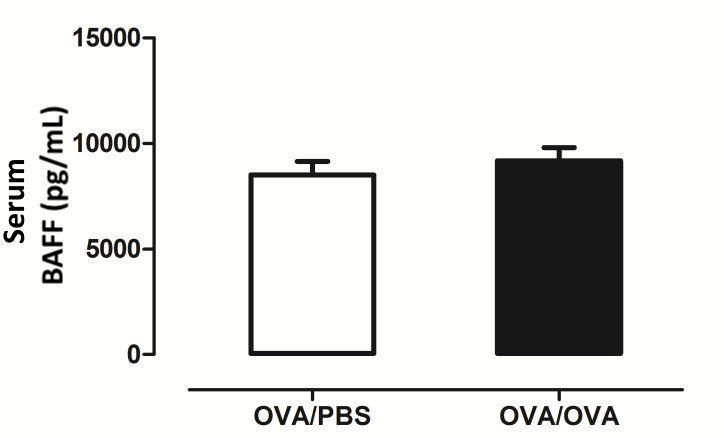

Supplement: S2 Fig — (TIF) [file pone.0161161.s002.tif]

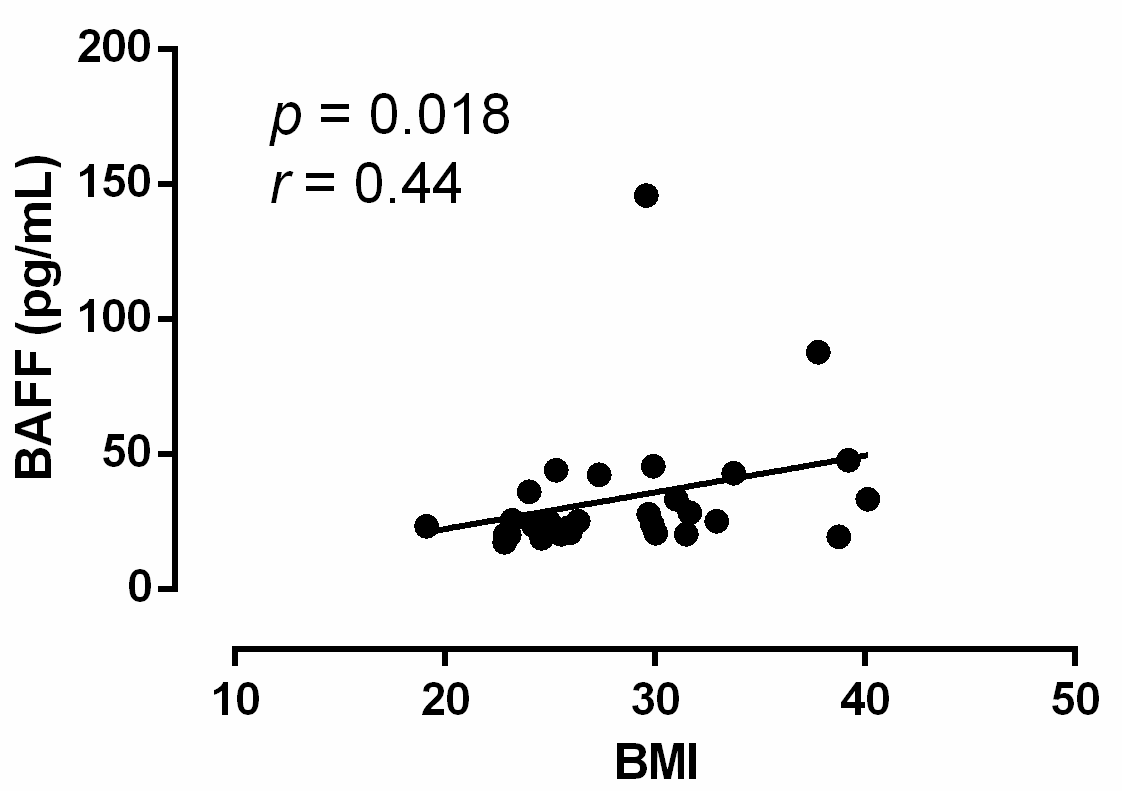

Supplement: S4 Fig — (TIF) [file pone.0161161.s004.tif]

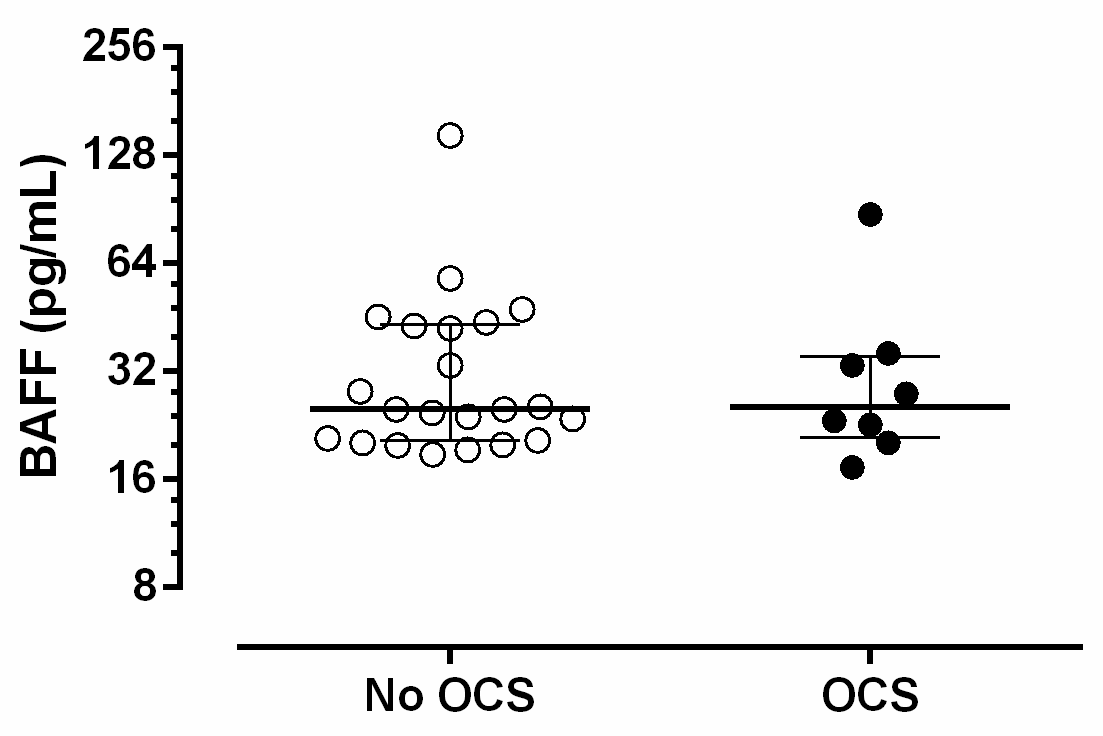

Supplement: S5 Fig — (TIF) [file pone.0161161.s005.tif]
