# Supplementary material for: Precursor B Cells Increase in the Lung during Airway Allergic Inflammation: A Role for B Cell-Activating Factor
Source: PLoS One. 2016 Aug 11;11(8):e0161161. doi: 10.1371/journal.pone.0161161 (PMC4981371; doi:10.1371/journal.pone.0161161)
Supplement: S1 Materials and Methods — (DOCX) [file pone.0161161.s006.docx]

**MATERIALS AND METHODS S1**

**Sample collection and processing**

Sample collection and processing was performed as previously described [1,2]. Samples were collected 24 h after the final OVA exposure. The animals were deeply anesthetized with a mixture of xylazin (130 mg/kg, Rompun^®^, Bayer, Germany) and ketamine (670 mg/kg, Ketalar^®^, Parke-Davis, England) and sacrificed by puncturing the right heart ventricle, at which point blood was collected. Mice were tracheotomized and bronchoalveolar lavage fluid (BALF) was performed by instilling 0.25 ml PBS through the tracheal cannula, followed by gentle aspiration and a second lavage with 0.20 ml PBS. An additional 1 ml of PBS was used to wash away airway lumen inflammatory cells, before the lungs were perfused and removed to harvest parenchymal inflammatory lung cells. The right apical lobe was stored at -80^o^C for subsequent protein analysis (ELISA) and the remaining three right lobes, without any connective tissue, were stored on ice in Hanks balanced salt solution (HBSS) (Sigma-Aldrich) before use. Bone marrow (BM) cells were harvested by excising one femur which was cut at the epiphysis and flushed with 2 ml HBSS. Samples of BALF and BM were centrifuged at 300 g for 10 min at 4° and the corresponding supernatants were saved for future measurements.

**Preparation of lung single-cell suspensions**

The right lung lobes were weighed and rinsed in a Petri dish before being transferred to a GentleMACS^TM^ C-Tube (Miltenyi Biotec GmbH, Bergisch Gladbach, Germany) containing 5.0 ml of HBSS supplemented with 10% fetal calf serum (FCS) (Sigma-Aldrich), 100 ml Collagenase D solution (final concentration 2 mg/ml) and 20 ml DNase I solution (final concentration 80 U/ml). The mouse lung was dissociated using a GentleMACS^TM^ Dissociator (Miltenyi Biotec) according to the manufacturer’s instructions. Lung cell pellets were collected washed in PBS supplemented with 10% FCS and the cells were saved for flow cytometry analysis. Cytospins were prepared and stained with May-Grünwald Giemsa. Total cell numbers were determined using standard haematological procedures. Cell differentiation in blood, BM, BALF and lungs was determined by counting 300–500 cells using a light microscope (Zeiss Axioplan 2, Carl Zeiss, Germany). Cells were identified using standard morphological criteria, such as nuclear morphology, cell size and cytoplasmic granulation, as previously described [1,2].

**Human subjects**

Bronchoalveolar lavage fluid (BALF) samples used for the determination of BAFF levels were acquired from a separate study, in which non-atopic healthy controls and patients with persistent mild-moderate (MMA) and severe asthma (SA) were recruited and underwent bronchoscopy. Study design and methods, as well as parts of the results are already published [3]. BALF samples were used from a total of 44 non-smoking subjects (14 healthy controls, 11 MMA and 19 SA). Informed and written consent was obtained from all participating subjects, and the corresponding protocol was approved by the Hospital Research Ethics Committee, as well as the Greek National Organization for Medicines (both Athens, Greece). Asthma was defined as a clear clinical history plus 15% reversibility in forced expiratory volume in one second (FEV_1_) after two puffs of β_2_–agonist and/or positive metacholine challenge. Atopy was assessed by means of skin-prick tests for 18 common aeroallergens (HAL Allergy Benelux, Leiden, the Netherlands). Participants showed no evidence of upper respiratory tract infection for at least two months prior to bronchoscopy. Patients were treated at the time according to GINA guidelines [4]. Healthy individuals had no history of asthma or any other chronic disease.

**Bronchoscopy and sampling**

Bronchoscopy was performed by an experienced bronchoscopist using a flexible bronchoscope (WM-N60 mobile workstation; Olympus, Tokyo, Japan) on an outpatient basis at the Athens Chest Hospital, as previously described [3,5]. After inspection of the bronchial tree, lavage was performed according to the European Respiratory Society Task Force guidelines [6]. Briefly, 100 mL of pre-warmed 0.9% saline was instilled into the right middle lobe in 20 mL aliquots and then gently aspirated with a recovery rate of ≥ 60%. The first aliquot collected was discarded. BALF samples were centrifuged for 15 min at 300 x g at 4^o^C and the supernatants were aliquoted and stored at -80^o^C within 2 h after collection until further use.

Reference List

1. Lu Y, Sjostrand M, Malmhall C, Radinger M, Jeurink P, Lotvall J, Bossios A (2010) New production of eosinophils and the corresponding TH1/TH2 balance in the lungs after allergen exposure in BALB/c and C57BL/6 mice. Scand J Immunol 71: 176-185. SJI2363 [pii];10.1111/j.1365-3083.2009.02363.x [doi].

2. Radinger M, Bossios A, Sjostrand M, Lu Y, Malmhall C, Dahlborn AK, Lee JJ, Lotvall J (2011) Local proliferation and mobilization of CCR3(+) CD34(+) eosinophil-lineage-committed cells in the lung. Immunology 132: 144-154. 10.1111/j.1365-2567.2010.03349.x [doi].

3. Samitas K, Zervas E, Xanthou G, Panoutsakopoulou V, Gaga M (2013) Osteopontin is increased in the bronchoalveolar lavage fluid and bronchial tissue of smoking asthmatics. Cytokine 61: 713-715. S1043-4666(13)00026-4 [pii];10.1016/j.cyto.2012.12.028 [doi].

4. [Anonymous]From the Global Strategy for Asthma Management and Prevention, Global Initiative for Asthma (GINA). NHLBI/WHO workshop Report. Updated 2009.

5. Zervas E, Samitas K, Vittorakis S, Koutsami M, Thomopoulos A, Liapikou A, Economidou E, Gaga M (2010) Safety of research bronchoscopy in mild-moderate and severe asthma. Pneumon 23: 41-47.

6. Haslam PL, Baughman RP (1999) Report of ERS Task Force: guidelines for measurement of acellular components and standardization of BAL. Eur Respir J 14: 245-248.
