# Supplementary material for: Precursor B Cells Increase in the Lung during Airway Allergic Inflammation: A Role for B Cell-Activating Factor
Source: PLoS One. 2016 Aug 11;11(8):e0161161. doi: 10.1371/journal.pone.0161161 (PMC4981371; doi:10.1371/journal.pone.0161161)
Supplement: S1 Table — (DOCX) [file pone.0161161.s007.docx]

**Table S1.** Antibodies used for Flow Cytometry

| **Antibody/Clone** | **Labeling** | **Company** |
| --- | --- | --- |
| **Surface Staining** | | |
| Anti-B220 (clone RA3-6B2) | PerCP, PE, AF-700, APC-H7 | BD Biosciences |
| Anti-CD93 (Clone AA4.1) | FITC |  |
| Anti-CD117/c-Kit (clone ACK45) | PE |  |
| Anti-CD43 (clone L448/S7) | PE, BV510 |  |
| Anti-CD127/IL-7Ra (clone SB/199) | PE, APC |  |
| Anti-CD184 (clone 2B11/CXCR4) | APC |  |
| Anti-CD40 (clone 1C10) | APC |  |
| Anti-BP-1 (clone 6C3/BP1) | FITC, (PE-Cy-7) | Biolegend |
| Anti-CD268/BAFFR (clone eBio /H22-E16) | AF-647 | eBioscience |
| Anti-CCR10 (clone 248918) | PE | R&D |
| Anti-BCMA ( Clone 161616) | FITC | R&D |
| Anti-TACI/CD267 (clone 8F10) | AF-647 | BD Pharmingen |
| Anti-69 (clone H1.273) | PerCP | Biolegend |
| Anti-CD86 (clone GL-1) | PerCP/Cy5.5 | Biolegend |
| **Intracellular Staining** | | |
| Anti-Bcl-2 (clone C-2) | AF-647 | Santa Cruz |
| Anti-Bax (clone 2D2) | PerCP/Cy5.5 | Santa Cruz |
| BrdU Flow Kits | FITC | BD Pharmingen |

Abbreviations

PerCP: Peridinin Chlorophyll Protein Complex, PE: PhycoErythrin, AF:

Alexa Fluor, FITC: Fluorescein isothiocyanate, APC: Allophycocyanin
