# Supplementary material for: Precursor B Cells Increase in the Lung during Airway Allergic Inflammation: A Role for B Cell-Activating Factor
Source: PLoS One. 2016 Aug 11;11(8):e0161161. doi: 10.1371/journal.pone.0161161 (PMC4981371; doi:10.1371/journal.pone.0161161)
Supplement: S2 Table — B cell precursor subsets as well as the markers used for their identification are highlighted in yellow. ** p < 0.01. (DOCX) [file pone.0161161.s008.docx]

**Table S2.** Stepwise differentiation of HSCs to immature B cells in the bone marrow, depicting the expression of cell-surface molecules according to their developmental stage and underlining the surface markers used in the flow cytometry to identify B cell subtypes. B cell precursor subsets as well as the markers used for their identification are highlighted in yellow. ** p < 0.01.

|  | **HSC** | **MPP** | **LMPP** | **ELP** | **CLP** | **Pre Pro B (Fr.A)** | **Early Pro B (Fr.B)** | **Late Pro B (Fr.C)** | **Large Pre B (Fr.C')** | **Small Pre B (Fr.D)** | **Immature (Fr.E)** |
| --- | --- | --- | --- | --- | --- | --- | --- | --- | --- | --- | --- |
| **Lin** | - | - | - | - | - |  |  |  |  |  |  |
| **SCA-1** | + | + | + | + | Low |  |  |  |  |  |  |
| **c-KIT** | + | + | + | + | Low | - | Low | Low | - | - | - |
| **Flt3** | - | - | + | + | + | + | - | - | - | - | - |
| **CD34** | - | + |  |  |  |  |  |  |  |  |  |
| **CD43** | + | + | + | + | + | + | + | + | +/- | - | - |
| **VCAM-1** | + | + | - | - |  |  |  |  |  |  |  |
| **IL-7R** |  |  | - | +/- | + | Low | Low/+ | + | + | + | - |
| **CD44** |  |  |  |  | + |  |  |  |  |  |  |
| **CD93** |  |  |  |  | + | + | + | + | + | + | + |
| **B220** |  |  |  |  |  | + | + | + | + | + | + |
| **CD19** |  |  |  |  |  | - | + | + | + | + | + |
| **CD24** |  |  |  |  |  | Low/- | + | + | ++ | ++ | ++ |
| **CD25** |  |  |  |  |  | - | - | +/- | + | + | - |
| **BP-1** |  |  |  |  |  | - | - | + | + | + | - |
| **IgM/IgD** |  |  |  |  |  |  |  |  |  | -/- | +/- |

HSC: Hematopoietic stem cell; MPP: Multipotent progenitor; LMPP: Lymphoid-primed multipotent progenitor; ELP: Early lymphoid progenitor; CLP: Common lymphoid progenitor

“+”: expression; “++” high expression, “-“no expression, “low” low expression and “+/-“ denotes that the marker could be either expressed or not.
